# Genetic dissection of complex traits using hierarchical biological knowledge

**DOI:** 10.1371/journal.pcbi.1009373

**Published:** 2021-09-17

**Authors:** Hidenori Tanaka, Jason F. Kreisberg, Trey Ideker

**Affiliations:** Department of Medicine, University of California San Diego, La Jolla, California, United States of America; Broad Institute of Harvard and MIT, UNITED STATES

## Abstract

Despite the growing constellation of genetic loci linked to common traits, these loci have yet to account for most heritable variation, and most act through poorly understood mechanisms. Recent machine learning (ML) systems have used hierarchical biological knowledge to associate genetic mutations with phenotypic outcomes, yielding substantial predictive power and mechanistic insight. Here, we use an ontology-guided ML system to map single nucleotide variants (SNVs) focusing on 6 classic phenotypic traits in natural yeast populations. The 29 identified loci are largely novel and account for ~17% of the phenotypic variance, versus <3% for standard genetic analysis. Representative results show that sensitivity to hydroxyurea is linked to SNVs in two alternative purine biosynthesis pathways, and that sensitivity to copper arises through failure to detoxify reactive oxygen species in fatty acid metabolism. This work demonstrates a knowledge-based approach to amplifying and interpreting signals in population genetic studies.

## Author summary

Genome-wide association studies (GWAS) have identified many important loci for common diseases and other traits. However, the loci identified by these studies are almost always many steps away from an understanding of underlying biological mechanisms. Here we develop an approach using hierarchical biological knowledge to identify genes and pathways responsible for phenotypic traits. Variants identified by the new method could explain a substantially greater fraction of heritability than previously reported. Moreover, we identified mechanistic pathways by which each causal variant affects cellular function. For example, we find that sensitivity to hydroxyurea is tied to genetic variants in two alternative purine biosynthesis pathways, and that sensitivity to copper arises through failure to detoxify reactive oxygen species in fatty acid metabolism. The new approach is a potentially transformative concept for understanding the genetic drivers of phenotypic variance, with potential applications in understanding traits in biomedicine and agriculture.

## Introduction

In recent decades, genome-wide association studies (GWAS) in humans have identified almost 19,000 associations between genetic loci and phenotypic traits [1]. In many cases though, the associated loci explain only a small fraction of the total heritable genetic variation suspected for that phenotype [2]. Of the various explanations put forward for this phenomenon, a frequently discussed possibility is that complex disease genetics are driven by large numbers of alleles, each with small effect sizes, making them hard to detect through genome-wide association [3]. To address this challenge, more complex models such as polygenic risk scores (PRS) have been developed, which sum effects across many variants to predict phenotype [4–6]. Even these more expansive models remain incomplete, in part because they do not account for non-linear interactions among variants [7].

A second major challenge for both GWAS and PRS is that these approaches do not provide clear insight into molecular mechanisms. Statistical associations between a genetic locus and a phenotypic trait have been typically difficult to translate to an understanding of which genetic variant(s) at that locus are causal, whether these variants affect the expression or activity of gene(s), and how these gene alterations give rise to variation in biological functions within cells and tissues. One way to identify potential links to candidate genes is to integrate orthogonal datasets, such as expression quantitative trait loci (eQTL), chromatin structure, or epigenetic marks [8–10]. As many of the variants identified by GWAS are located in noncoding regions, follow-up experiments typically entail reporter assays, validations of transcription factor binding sides, animal models and genome engineering [11–13]. Even these techniques do not begin to address functional effects of the variant beyond the gene, such as impacts on the states of proteins, protein complexes, metabolic processes and signaling pathways, and composition of cell types. Thus, the process of translating an associated locus to a causal single nucleotide variant (SNV) and then to a causal gene and subsequent underlying biological mechanism is still far from routine.

To address these challenges, a growing field of approaches has begun to look for causal and mechanistic links not in individual genes, but among groups of genes that are functionally related by pathways or gene networks [14–18]. Called Pathway GWAS or Network GWAS, these methods share the intuition that prior knowledge can increase statistical power and interpretability of genetic analysis, by pooling signals across sets of genes organized by common functions [19–23]. A benefit of these approaches is that they can greatly reduce the number of hypotheses tested, since the number of genes and gene sets is substantially fewer than the number of SNVs. In addition, these approaches simplify functional interpretation, since genotypes are connected to phenotypes *via* their effects on core biological functions.

Related to these approaches, we recently developed a supervised machine learning system for predicting the phenotypic outcome of genetic mutations using the method of “ontotypes” [24]. In the ontotypes approach, disruptions to genotype are first translated to disruptions in cellular systems at multiple scales, based on prior knowledge encoded by an ontology of cell structures and functions such as the Gene Ontology (GO) [25]. These ontotypes are then used as engineered features to train a supervised machine learning algorithm to predict phenotypic outcomes. In addition, unlike black-box machine learning approaches, the ontology features can be readily interpreted to yield mechanistic insights and visualizations of genotype-ontotype-phenotype relationships [24,26]. For example, ontotypes revealed previously unknown connections between intron homing and the phosphatidylinositol-3-kinase complex as well as between the tubulin complex assembly and DNA-directed RNA polymerase I [24]. These and other findings demonstrated that ontoypes could be used for predicting and understanding the underlying molecular mechanisms by which gene disruptions affect phenotype. Furthermore, the ontology of cellular systems need not be drawn from literature but can also be inferred directly from various sources of data [27,28].

Here, we explore whether a similar ontotype methodology can be applied to decode genome-wide association studies. As a test-bed for exploration, we consider a recent GWAS that sequenced over 1000 isolates of *Saccharomyces cerevisiae* and, in parallel, phenotyped these isolates across 36 growth conditions [29]. The initial analysis of these data mapped 35 genetic variants associated with 14 growth phenotypes [29]. In many of these cases, the molecular mechanisms by which the variants modulate phenotype were unclear, and the study noted large gaps between the amount of phenotypic variance explained and the total estimated genome-wide heritability.

To achieve greater coverage and mechanistic insight in how genetic variation leads to phenotypic variation in yeast, we now analyze the same collection of genetic isolates with a knowledge-based ontotypes approach. This analysis identifies a constellation of biological systems driving genotype-phenotype predictions, including the discovery that genetic variants in glycine cleavage play major roles in the response to genotoxic stress by hydroxyurea (HU). In nearly all cases, the genetic variants and associated biological systems identified by ontotype analysis have not been previously discovered by standard gene association tests.

## Results

### Mapping causal variants using ontotypes

**Fig 1:**
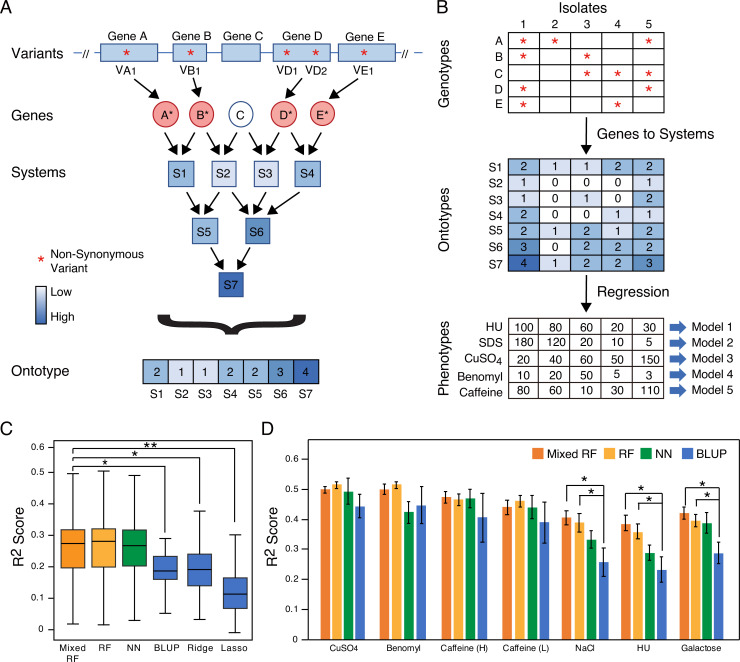
Overview of Ontology-Guided Learning. (A) Genetic variants (non-synonymous SNVs, red asterisks) propagate hierarchically from potential effects on genes to small gene systems such as protein complexes to broad processes and organelles. The “ontotype” represents the comprehensive vector of effects across this hierarchy of systems. (B) System values in ontotype are regressed against phenotype using a non-linear random forests model. Red asterisks indicate genetic variants; phenotype scores are the area of the yeast colony measured in pixels. CuSO_4_, copper sulfate. HU, hydroxyurea. SDS, sodium dodecyl sulfate. (C) Accuracy of phenotypic prediction (R^2^ score over 36 traits, y axis) using ontotypes as inputs for mixed random forest models (Mixed RF; dark orange bar), random forest models (RF; light orange bars), neural networks (NN; green bars) or three linear models (blue bars). (D) R^2^ values for each of seven individual traits showing averages over five-fold cross-validation. Mixed random forest, random forest, neural networks and BLUP models are shown (dark orange, light orange, green and blue bars, respectively). * *P* < 0.05, ** *P* < 0.001 by two-tailed unpaired t-test with Welch correction. Caffeine (H) and (L) correspond to 50 and 40 mM respectively.

We explored mapping of causal variants from GWAS using genotype-phenotype data previously gathered in approximately 1000 natural *S*. *cerevisiae* isolates [29]. Specifically, we focused on 18,931 SNVs that correspond to non-synonymous substitutions in coding regions (Fig 1A; Variants). For representing individual genotypes, any gene harboring one or more non-synonymous SNVs was scored a “1” with the rest scored a “0” (Fig 1A; Genes). To translate genotype to an ontotype for every isolate, we counted the number of genes with non-synonymous SNVs in each biological system (Fig 1A and 1B; Systems). As biological systems for these initial studies, we used the ontology of biological processes, cellular components, and molecular functions present in GO. The resulting vector of system scores defined the ontotype for each isolate (Fig 1A and 1B; Ontotype).

One challenging aspect of population genetics is accounting for population structure. Here, we first sought to compare random forest models, which were used successfully in an earlier study using ontotypes [24], with “mixed” random models, a modified version of random forest models that can account for both population structure and nonlinear interactions when analyzing GWAS data [30]. When trained using ontotypes for each of the 36 traits [29], both random forest models and mixed random forest models yielded similar coefficients of determination, R^2^ values (Figs 1C, 1D and S1A), suggesting that the overall fits of both models were similar. In addition, the sets of most important systems identified by each model were very similar (S1B and S1C Fig, **Materials and Methods**). With both models generating similar results, we chose to use the more conservative, established approach and focused our efforts on the results from the mixed random forest models. R^2^ scores for six traits – copper sulfate, benomyl, caffeine, sodium chloride (NaCl), HU and galactose – were notably higher than the others with values near or greater than 0.4 (Fig 1D, henceforth called *well-predicted traits*).

We next compared these R^2^ scores to those of models using ontotypes as inputs but trained using neural networks or various linear prediction methods: ridge regression, lasso regression or best linear unbiased prediction (BLUP), a linear mixed model [31]. The R^2^ values from the neural network models were similar to both the mixed random forest models and random forest models (Figs 1C, 1D and S1D). Across the 36 phenotypes, the R^2^ scores for the mixed random forest models were significantly higher than those of BLUP (*P* = 0.012, two-tailed unpaired t-test with Welch’s correction; Fig 1C), ridge regression (*P* = 0.031, two-tailed unpaired t-test with Welch’s correction; Fig 1C) and lasso regression (*P* = 6 x 10^-6^, two-tailed unpaired t-test with Welch’s correction; Fig 1C). Similar trends were seen when focusing on specific traits, with R^2^ scores from mixed random forest models significantly higher than BLUP in traits such as NaCl, HU and galactose (*P* < 0.05, two-tailed unpaired t-test with Welch’s correction; Fig 1D). These data demonstrated that the mixed random forest method can outperform linear models when using ontotypes for phenotypic prediction.

### Ontotype models identify novel genes focused within compact systems

To test the importance of using ontotypes as input features, we instead used SNVs or genes to build mixed random forest models for each of the 36 phenotypes. For the SNV-based models, we used the same set of 18,931 coding, non-synonymous SNVs that were used when constructing ontotypes; each SNV was scored as a “0” for the reference allele or a “1” for a variant. For the gene-based models, genes were scored a “0” if all of the coding region SNVs correspond to the reference alleles and a “1” if otherwise (Fig 1A). To evaluate these three different sets of features – ontotypes, genes or SNVs – for training mixed random forest models, we compared the predictive performance across traits and found that the performance of each set of features was roughly equivalent across models (S2 Fig).

**Fig 2:**
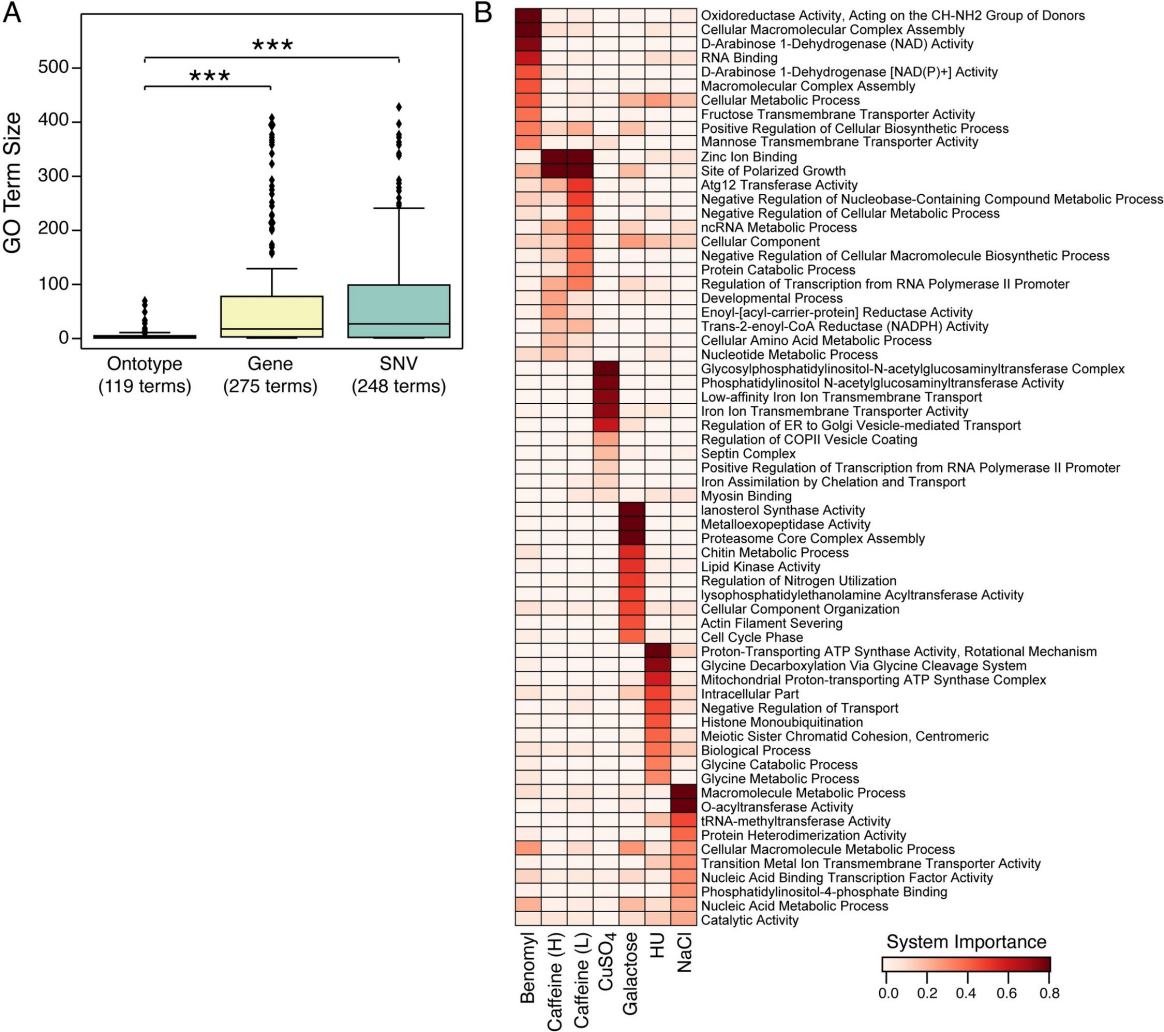
Important Genes and Systems in Phenotypic Prediction. (A) The sets of genes identified by SNV- or gene-based models for copper sensitivity were translated to systems using GO enrichment analysis. The boxplots show the size distributions of these systems in comparison to systems identified directly by features in ontotype analysis. *** *P <* 0.01, two-tailed unpaired t-test with Welch correction. (B) Heatmap showing importance of systems (rows) across traits (columns). The top ten features from each model are shown. Feature importance score was normalized to range from 0 (white) to 1 (red). Caffeine(H) and (L) correspond to 50 and 40 mM respectively.

We then compared the systems (GO terms) identified by the ontotype-based mixed random forest models to those identified by the SNV- or gene-based mixed random forest models (based on GO enrichment analysis, **Materials and Methods**). Notably, ontotype models identified fewer, more focused sets of systems than other approaches (Fig 2A for copper and S3 Fig for the others; S1 Table). The vast majority of important systems for each phenotype were unique to that phenotype (Fig 2B), with the exception of systems identified for different doses of caffeine, which overlapped nearly completely (positive control). Thus, ontotypes capture a wide range of systems underlying phenotypic diversity.

### Other systems-based approaches

We also attempted to use two other approaches – GSA-SNP2 [23] and Gowinda [32] – to analyze the yeast phenotype data on copper, benomyl and 40 mM caffeine, three of the well-predicted traits. Both of these approaches use *P* values determined from a conventional GWAS study to assign *P* values to GO terms, a two-step process. Notably, we found that neither approach identified any GO terms with *P* < 0.05 for any of the three phenotypes. In contrast, our ontotype-based approach with an empirical *P* < 0.01 identified 119 terms for copper at an empirical false discovery rate (FDR) of 0.0084 (**Materials and Methods** for details on calculating empirical *P* values and FDRs), 64 terms for benomyl (0.031 empirical FDR) and 47 terms for 40 mM caffeine (0.043 empirical FDR). For the other well-predicted traits, we found 42 terms for 50 mM caffeine (0.048 empirical FDR), 59 terms for HU (0.017 empirical FDR) and 30 terms for galactose (0.033 empirical FDR). These findings suggest that our ontotype models can detect genetic associations based on the convergent effects of many SNVs, even when the marginal effects of each of these SNVs may not be significant.

### Copper toxicity is regulated by intracellular vesicle transport

**Fig 3:**
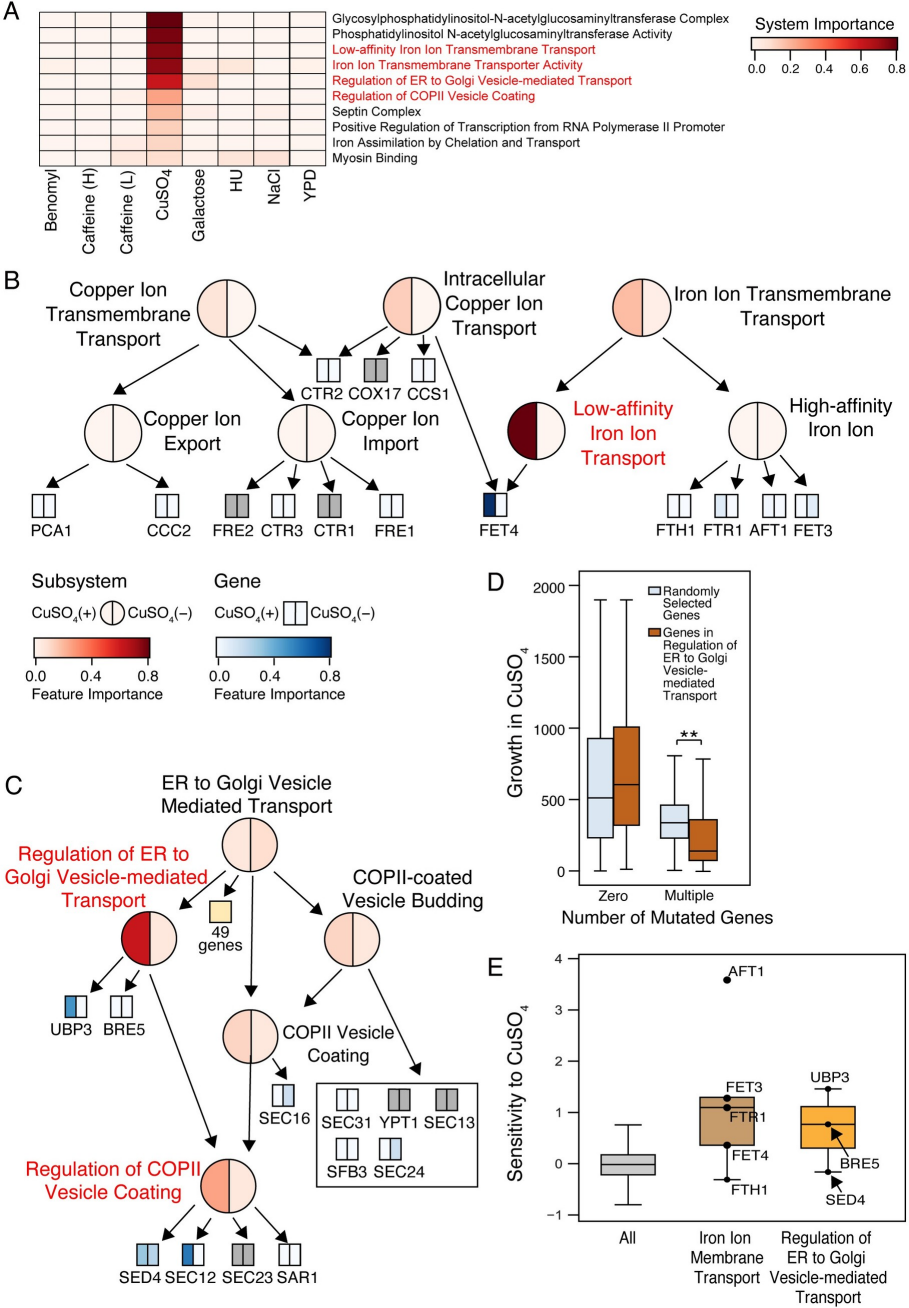
Genetics and Mechanisms of Copper Sensitivity. (A) Heatmap showing importance of the ten most important systems (rows) in predicting copper response. Feature importance score was normalized to range from 0 (white) to 1 (red). (B) Subhierarchy of systems (represented as circles) and genes (represented as squares) related to copper transport. The intensity of red and blue indicates the importance of systems and genes, respectively, after treatment with copper on the left half versus a control treatment on the right half. Systems with red labels are among the top ten important features for prediction. Gray squares indicate genes without non-synonymous SNVs. (C) Subhierarchy of systems and genes within the ER-to-Golgi Vesicle Mediated Transport system. Similar to B. (D) Boxplots showing the relationship between yeast growth (colony area in pixels) and the number of mutated genes in the “regulation of ER to Golgi vesicle-mediated transport” system (brown) compared to the same number of randomly selected genes (blue). The random process was repeated 1000 times. * *P* < 0.05 and ** *P* < 0.001 from a one-way ANOVA test with Tukey correction for multiple comparisons. (E) Copper sensitivity in targeted single gene deletion mutants, shown for all non-essential yeast genes (left) and genes in systems detailed in panels B (middle) and C (right). Sensitivity depicts log2 ratios of growth between 500 μM CuSO_4_-treated and -untreated samples. High sensitivity values indicate gene knockouts that had a negative effect on growth.

Copper is an essential trace element that is important for processes such as respiration and protein modification. High doses can be toxic though due to hydroxyl radical formation that directly damages DNA, membrane lipids and proteins. The ontotype model identified the systems “low-affinity iron ion transmembrane transport” and “iron ion transmembrane transporter activity” as two of the most important systems for predicting growth in copper sulfate (Fig 3A). Both systems contain the metal transporter *FET4* (Fig 3B), suggesting that variants of *FET4* impact copper sensitivity. Among other highly ranked systems were “regulation of ER to Golgi vesicle-mediated transport” and “regulation of COPII vesicle coating” (Fig 3A and 3C). Strains with multiple non-synonymous SNVs in genes assigned to “regulation of ER to Golgi vesicle-mediated transport” were more sensitive to copper sulfate than strains without any mutations (Fig 3D), consistent with these genes playing an important role in this phenotype.

Further support for these pathways was provided by two independent datasets. Knockouts of genes [33] functioning in “iron ion transmembrane transport,” an expected function related to copper, and “regulation of ER to Golgi vesicle-mediated transport,” an entirely unexpected finding, significantly increased copper sensitivity (Fig 3E). Another previous report [34] found that disrupting *FET4* in the reference strain S288C renders cells sensitive to copper toxicity. Genes in the “regulation of ER to Golgi vesicle-mediated transport” pathway such as *UBP3*, *BRE5* and *SED4* have not previously been reported to impact copper sensitivity. Collectively, these findings demonstrate that ontotype models can not only shed light on well-established pathways but also identify novel functions.

### Glycine metabolism impacts response to HU

**Fig 4:**
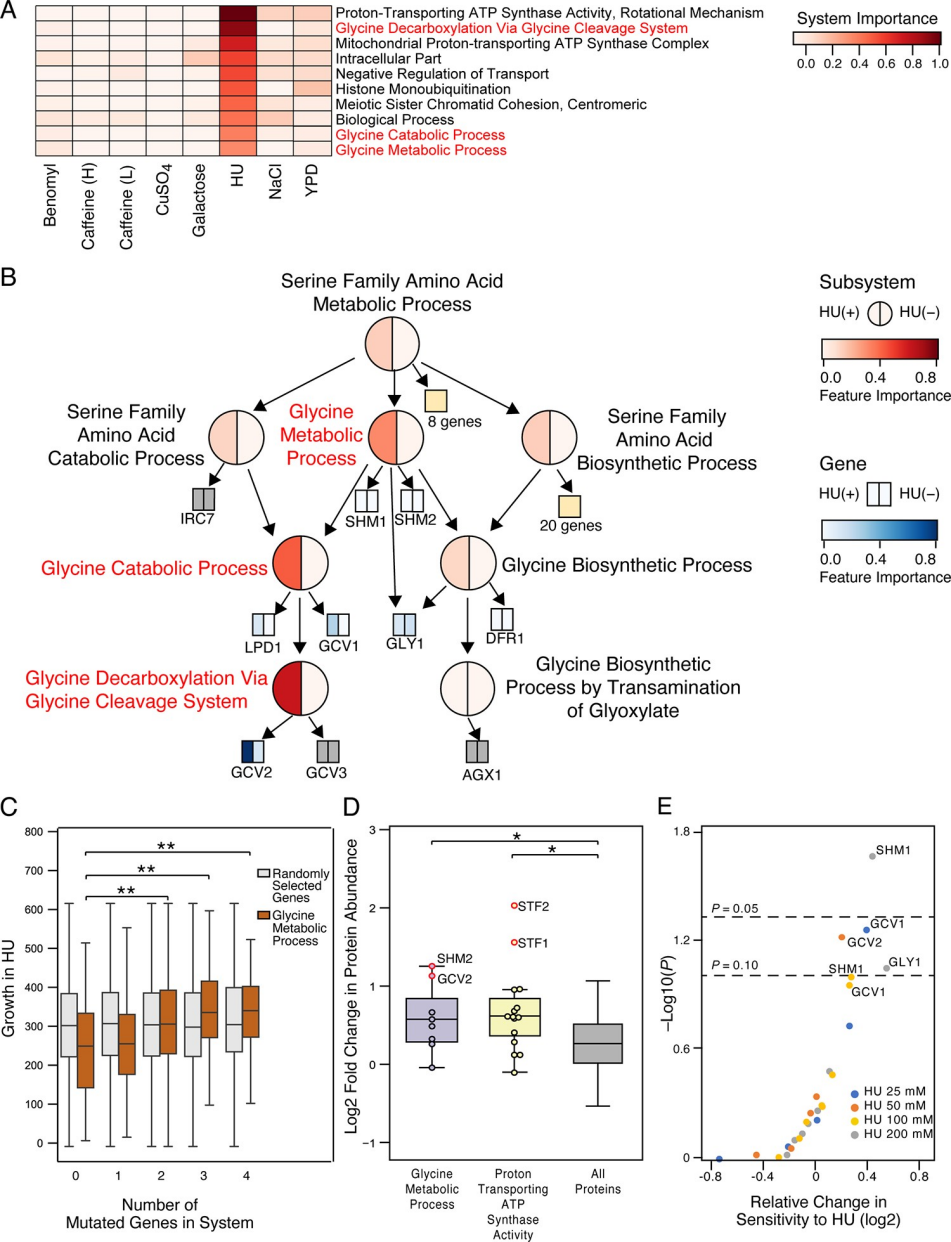
Genetics and Mechanisms of Sensitivity to HU. (A) Heatmap showing importance of the ten most important systems in predicting HU response. Feature importance score was normalized to range from 0 (white) to 1 (red). (B) Subhierarchy of systems and genes within “serine family amino acid metabolic process.” Layout similar to Fig 3B but with feature importance for HU treatment on the left half and for control treatment on the right half. (C) Boxplots showing the relationship between yeast growth (colony area in pixels) and number of mutated genes in “glycine metabolic process” (brown) compared to the same number of randomly selected genes (grey). ** *P* < 0.001 from a one-way ANOVA test with Tukey correction for multiple comparisons. (D) Boxplots showing the relative change in protein abundance in response to 120 mM HU. Positive scores indicate increasing protein levels. Red circles indicate genes within each system that are significantly increased when compared to all proteins (*P* < 0.01, *z*-test). * *P* < 0.05 and ** *P* < 0.001 from one-way ANOVA tests with Tukey correction for multiple comparisons. (E) Relative change in sensitivity to HU in targeted single gene deletion mutants versus wild type. Plotted for each single gene deletion are the log2 ratios of growth under normal conditions relative to growth when treated with HU.

HU is used to treat certain types of cancer and to reduce the need for blood transfusions in people with sickle cell anemia. HU functions by inhibiting ribonucleotide reductase activity resulting in the suppression of DNA synthesis [35]. Ontotype analysis of HU response in yeast identified systems such as “glycine decarboxylation via glycine cleavage system” and “proton transporting ATP synthase activity” as playing key roles (Fig 4A). Taking a closer look at the systems near “glycine decarboxylation via glycine cleavage system,” we found two related systems that also scored highly: “glycine catabolic process” and “glycine metabolic process” (Fig 4A and 4B). An increased mutational burden in “glycine metabolic process” was associated with increased growth in the presence of HU (Fig 4C).

To further explore a role for these genes and systems when responding to HU, we analyzed two independent datasets. In the first, yeast were exposed to HU and proteome-wide expression levels were measured [36]. Here we found that protein expression levels for the key systems identified by ontotypes were significantly increased in response to HU exposure (*P* < 0.05; Fig 4D). Within these systems, the protein expression levels of SMH2, GCV2, STF2 and STF1 increased by 2- to 4-fold (*P* < 0.01; Fig 4D). Using a second dataset where the single gene knockouts were tested for their sensitivity to HU [37], we found that *SHM1* plays a significant role (*P* < 0.05; Fig 4E) and that *GCV1*, *GCV2* and *GLY1* are statistically borderline (*P* < 0.1; Fig 4E) with *GLY1* knockouts having the largest effect size (Fig 4E). As these gene knockouts rendered yeast more sensitive to HU whereas naturally occurring isolates with more mutations in these genes were less sensitive, these naturally occurring variants may correspond to gain-of-function mutations.

### Glucose transport plays a central role in benomyl sensitivity

Benomyl is toxic to *S*. *cerevisiae* and other microorganisms by destabilizing microtubules leading to spindle depolymerization and cell cycle arrest [38]. Ontotype analysis identified a cluster of related systems – “glucose transmembrane transporter activity,” “mannose transmembrane transporter activity” and “fructose transmembrane transporter activity” – as playing key roles in modulating sensitivity to benomyl (S4A and S4B Fig). The mutational burden in “glucose transmembrane transporter activity” was correlated with decreased growth (i.e., increased sensitivity, S4C Fig).

### Discovery of novel biological systems using a data-driven ontology

While the above analysis was able to successfully identify and interpret many new genetic associations, it was based on recorded knowledge of cellular systems, which is limited. To permit the identification of new biological systems during GWAS, we replaced GO with an ontology of cellular subsystems derived directly from yeast ‘omics data [24,27,28] (**Materials and Methods**). Across all phenotypes, the distribution of R^2^ values from ontotype-based models using a data-derived hierarchy was similar to that of ontotype-based models using GO terms (S5 Fig).

**Fig 5:**
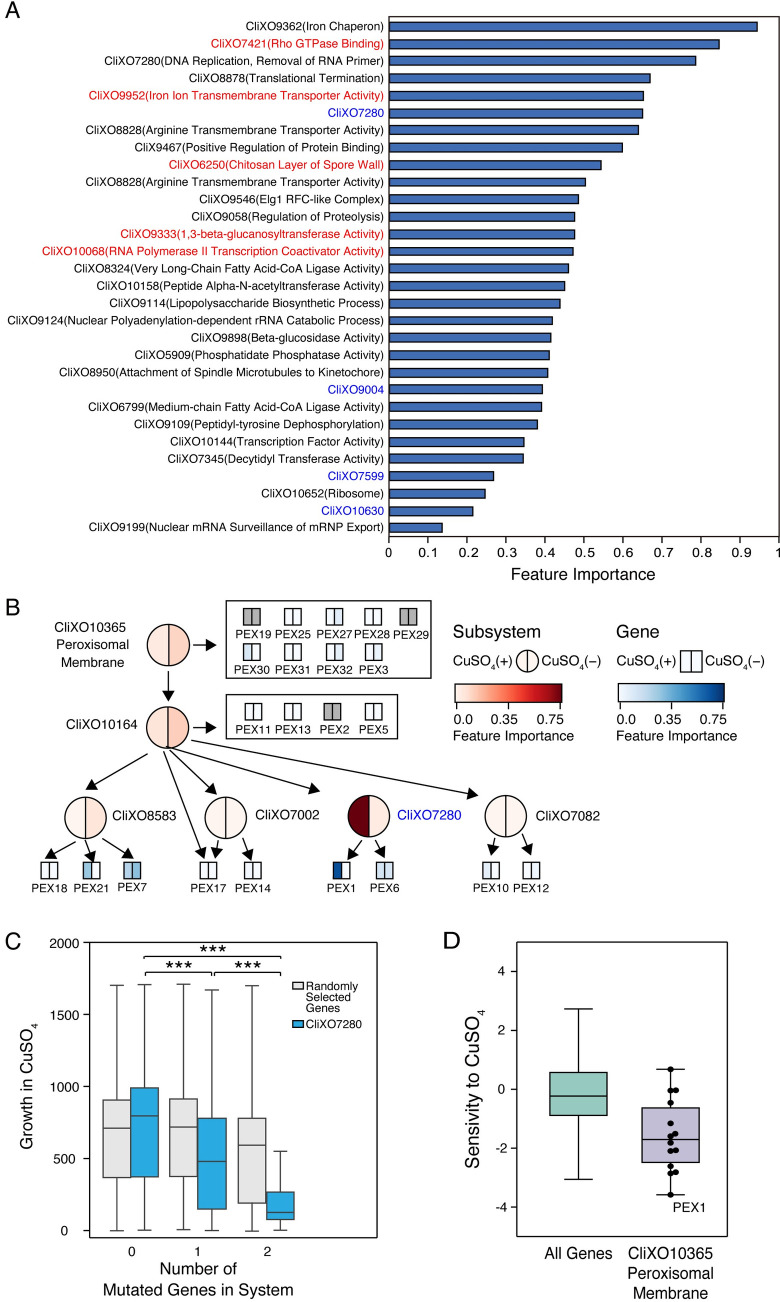
Computing Ontotypes with a Data-Driven Systems Ontology. (A) Bar graph of importance scores for the 30 most important data-derived systems in copper response, normalized from 0 to 1. Blue labels indicate data-derived systems that do not align with any literature-derived term in GO. Red labels indicate systems whose aligned GO terms are in the top thirty most important previously identified (Fig 3). (B) Hierarchy of systems and genes within the peroxisomal membrane. Layout similar to Fig 3B. (C) Relationship between yeast growth and the number of mutated genes in novel system CliXO7280 (blue) compared to the same number of randomly selected genes (grey). *** *P* < 1 x 10^−9^, one-way ANOVA with Tukey multiple comparison test. (D) Copper sensitivity in single gene deletion mutants for all genes versus the gene set in the peroxisomal membrane.

The new ontotype-based model for growth in copper had an R^2^ value of 0.49 (S5C Fig) with 5 of the 30 most predictive data-derived systems implicating similar sets of genes to the top GO terms identified previously (Fig 5A, red colored systems). Highlighting potentially novel findings, 4 of the 30 most predictive data-derived systems contained genes that did not align well with any current GO terms (Fig 5A, blue colored systems). For example, CliXO7280, the sixth most important data-derived system, captured a cluster of genes within the peroxisomal membrane (CliXO10365, Fig 5B) corresponding to a heterodimeric complex of two AAA-peroxins PEX1 and PEX6 required for the biogenesis of peroxisomes [39]. Peroxisomes were not identified using ontotypes based on GO terms (Fig 3A), perhaps because the closest GO terms are much larger and less specific than the systems in the data-driven hierarchy, thereby diluting the genetic signal. The mutational burden in this system was correlated with growth sensitivity to copper (Fig 5C). Single gene knockouts of many of the genes in CliXO10365 (peroxisomal membrane) were found to cause copper sensitivity, with *pex1Δ* being the most sensitive (Fig 5D). Other top systems were involved in fatty acid-CoA ligation (S6A and S6B Fig), a subprocess within fatty acid beta-oxidization reactions which are restricted to peroxisomes. Overall these findings strongly support peroxisomes as a key mechanism in how cells respond to copper.

Our data-driven ontotype-based model for HU sensitivity identified three key systems that are closely related: CliXO7509, CliXO8419 and CliXO9394 (S7A Fig). In this case, growth was associated with the mutational burden in the most inclusive system (CliXO9394; S7B Fig). Our findings are consistent with a previous study [37] showing that single gene deletions of *MPC1* or *MPC2* are sensitive to HU (S7C Fig).

### Ontotype for GWAS explains a portion of missing heritability

**Fig 6:**
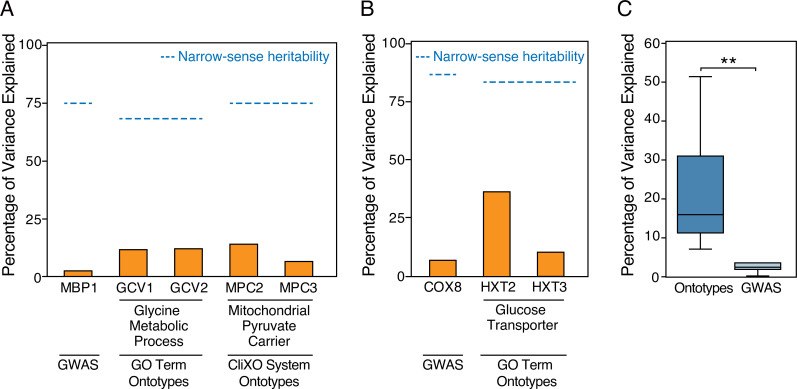
Phenotypic Variation Explained for each Model and Trait. (A–B) Percentage of variance in the growth phenotype (y axis) that is explained by key genes (x axis, orange bar) for predicting sensitivity to HU (A) or benomyl (B). Dashed lines represent the narrow-sense heritability specific to that model and trait. Data for MBP1 and COX8 are included as they were identified as important genes for these phenotypes in a previous study [29]. (C) Comparison of explained phenotypic variation between ontotypes and standard GWAS. ** *P* = 8 x 10^−3^, two-tailed unpaired t-test with Welch correction.

The original yeast GWAS for the data used here reported 35 genetic variants (including copy number variations and SNVs) associated with 14 growth conditions [29]. For many of these phenotypes, there was a large gap between the phenotypic variance explained by these variants and the overall narrow-sense heritability, which was much greater. Even though ontotype models were trained on a smaller set of SNVs than GWAS and based on mixed random forest models instead of linear mixed models, the narrow-sense heritability captured by the ontotype models was similar to GWAS (Fig 6A and 6B and S2 Table). We thus sought to compare variants identified for each trait by the ontotype approach to variants identified by the original GWAS on the same dataset. For example, for HU response, we found that the glycine metabolic genes (GCV1 and GCV2) and mitochondrial pyruvate carrier genes (MPC2 and MPC3) identified by ontotype explained a larger proportion of trait variance than the previously identified SNV in MBP1 (Fig 6A). Similarly for benomyl, the glucose transporter genes (HXT2 and HXT3) identified by ontotype explained more of the phenotypic variance in the benomyl response than the previously identified SNV in COX8 (Fig 6B). To identify the genes most responsible for explaining heritability in the ontotype models, we analyzed the collective contribution of genes present in the ten most important systems (filtered to retain the 29 most important genes overall; S3 Table). Overall, these 29 variants identified by ontotype models explained a larger proportion of trait variance compared to all SNVs identified as genome-wide significant in the previous GWAS study (*P* = 8 x 10^−3^, two-tailed unpaired t-test with Welch’s correction; Fig 6C). Thus, the variants identified by ontotype serve to fill some of the gap between narrow-sense heritability and explained phenotypic variance that is not accounted for by conventional GWAS.

## Discussion

Here we have seen that ontotype models can reveal the genes and systems underlying classic yeast growth phenotypes, and that in certain cases, these models provide more explanatory power than standard GWAS approaches. To transform an ontology into a multi-scale model capable of genotype-phenotype inference, we used an intuitive and parameter-free approach that simply determines mutational burden at the systems level. These ontotypes are then used as input features to train machine learning models for predicting phenotypes. The resulting ontotype-based models are readily interpretable, having recovered known mechanisms for certain phenotypes while suggesting novel functional roles in others.

The intuition behind a systems-based genetic approach, including ours here and others [14–23], is to treat the system itself as the basic genetic unit, rather than the SNV or gene. Our approach differs from previous ones in two key aspects. First, we build a single predictive model of phenotype capturing the combined effects of all genetic variants, in contrast to most previous approaches which test each genetic variant independently. Second, our approach seeks to directly capture the principal genetic effects of each system in one step. Other system-based approaches typically perform a two-step analysis where association statistics at the SNV level are computed first and then these values are used to analyze gene sets. Further studies need to be performed but this feature could be a key reason why our method can identify genetic signals that some other methods miss.

Yeast are equipped with a variety of defense mechanisms against toxic doses of copper such as chelators, exporters and scavengers of the oxygen free radicals generated by copper-mediated reactions [40]. Our GO-based ontotype model of copper sensitivity identified “ER to Golgi vesicle mediated transport” and related subsystems as important features. The negative association between growth and the number of SNVs in “ER to Golgi vesicle mediated transport” genes suggests that these mutations act as losses-of-function. Although metal ions are trafficked to extracellular proteins through a late or post-Golgi compartment in the secretory pathway [41], a specific connection between copper detoxification and secretory pathways has not previously been described in yeast.

Our complimentary study of copper sensitivity using a data-driven ontology also identified a nested set of systems related to peroxisomes as playing a key role. Peroxisomes are cytoplasmic organelles surrounded by a single lipid bilayer. Although some proteins are delivered to the peroxisome by the ER, many others, including the ROS detoxification enzyme catalase A, are imported directly from the cytoplasm. In yeast, peroxisomes are also the sole site of fatty acid beta-oxidation and consequently a source of reactive oxygen species that can damage nucleic acids, proteins and lipids. One of the top data-derived systems consisted of two genes: *PEX1* and *PEX6*. The proteins encoded by these two genes form a hexameric complex consisting of three subunits of each. This complex plays a key role in the import of many proteins into peroxisomes [42,43]. Another important system, CliXO6799, consisted of two genes: *PEX1* (again) and *FAA2*. *FAA2* is a peroxisome-localized enzyme involved in fatty acid activation and import [44]. The primary source of hydrogen peroxide, an especially potent ROS, is fatty acid beta-oxidation in the peroxisome [44]. Our results suggest that peroxisome dysfunction plays an important role in regulating copper sensitivity, perhaps due to defective import of detoxification proteins or ROS-production triggered by aberrant beta-oxidation processes.

HU inhibits ribonucleotide reductase leading to reduced levels of dNTPs (especially purines), inhibition of DNA synthesis and suppression of cellular proliferation. Depleted levels of intracellular dNTPs also lead to defective repair of single-strand DNA breaks. Our study of HU response identified as important features “glycine metabolic process” and its subsystems “glycine catabolic process” and “glycine decarboxylation via glycine cleavage.” The glycine cleavage system catabolizes glycine into 5,10-methylenetetrahydrofolate (5,10-CH2-THF), an essential precursor for DNA synthesis [45]. Glycine itself plays an important role in *de novo* purine synthesis through direct incorporation into the purine backbone for cancer proliferation [46]. Our findings suggest that the decreased levels of purines after HU treatment may be compensated by glycine decarboxylation and various THFs.

We also identified a pair of HU-response systems encompassing the genes *MPC1*, *MPC2* and *MPC3*. The protein products encoded by these genes form the mitochondrial pyruvate carrier (MPC), which transports pyruvate from the cytoplasm into the mitochondria where it can enter the tricarboxylic acid cycle [47,48]. Recently, it was reported in yeast that the DNA damage response activates respiration, which increases dNTP abundance to enhance cell survival [49]. Taking into consideration the relationship between the number of mutations and growth (S7 Fig), variants in these genes may improve respiratory activity through increased MPC function.

One shortcoming of the current approach is that it only takes into account non-synonymous mutations, thereby ignoring all linked regulatory variation. In addition, the assignment of non-synonymous SNVs to genes does not take into account linkage disequilibrium. This could lead to erroneous gene scores as nearby SNVs may be inherited on the same haplotypes as the non-synonymous SNVs used in the model. Ultimately, the contribution of other variants such as SNVs in non-coding regions and copy number variations should be evaluated in phenotypic prediction. Another potential improvement would be a refined approach to scoring genes. Rather than the binary “wild type” or “mutated” states used here, a revised quantitative score might use information about known or predicted severity of variants.

Testing different machine learning approaches could also be a fruitful avenue for future studies. Here we found that ontotype-based random forest models (mixed or otherwise) resulted in similar R^2^ values as did fully connected neural networks. In two previous studies though, we found that neural networks performed better than other machine learning approaches, even in one case when sophisticated feature engineering was performed [50,51]. Collectively, these findings suggest that feature engineering based on biological knowledge coupled with traditional machine learning methods can at times result in models with similar performance to fully connected neural networks. One challenge though of fully connected neural networks is that they are black boxes, exclusively focused on predicting outputs from inputs without regard for the mechanism or rationale by which a particular outcome is brought about [26]. As one path towards explainable artificial intelligence, our lab has made progress in “visible” machine learning, an approach that integrates the structure of genotype-phenotype statistical models with expansive knowledge of molecular mechanisms [50,51]. One notable challenge of this approach is to construct a high performing and informative underlying neural architecture; the size and connectivity of the mechanistically-informed network needs to be tuned in part on the amount of available training data.

In summary, our ontotype methods for decoding GWAS have revealed new biological insights into the underlying mechanism of a number of phenotypes in yeast. It is notable that a simple mutation counting approach, using either GO or a data-driven ontology, could be used to generate predictive models with mechanistic insights, especially as these models have been trained on far fewer features than a typical GWAS model. As ontologies such as GO are popular and widespread in other organisms, the methods described here could be readily applied to tackle clinical or agricultural traits.

## Materials and Methods

### Construction of SNV matrix used in this study

SNV data is available from the 1002 Yeast Genomes Project website (http://1002genomes.u-strasbg.fr/files/). The matrix used for the GWAS analysis (1011GWASMatrix.tar.gz) included files in BED (.bed), BIM (.bim), and FAM (.fam) formats. After piling up these formats by PLINK [52], reference sequence and altered sequence information was added from 1011Matrix.gvcf.gz, which contains all SNVs called at the population level.

### Annotation for each SNV

SnpEff was used for annotating the variants [53]. The final set of 18,846 non-synonymous SNVs (“missense” or “nonsense” in SnpEff) were labeled “SNV matrix.”

### Transformation of SNV matrix into gene matrix

The “SNV matrix” was converted into the “gene matrix” using an OR gate, meaning the maximum score for any gene is 1 even if that gene contains multiple non-synonymous mutations. The resulting “gene matrix” included information on 4,071 genes.

### Transformation of gene matrix into ontotype matrix

Files with GO structure and gene-to-term annotations were downloaded on December 19, 2011, from http://geneontology.org/ and correspond to the same structure as used in a previous study from the lab [24]. All three branches of the GO – biological process (BP), molecular function (MF) and cellular component (CC) – were used by joining them under a single root. In addition, terms that were not annotated with any yeast genes or that were redundant with respect to their children terms were removed. In total, 5,124 terms were drawn from all branches of GO: 707 terms from CC, 2,598 terms from BP and 1,819 terms from MF.

To construct a data-driven ontology, 68 networks including data about protein-protein interactions, gene co-expression and gene co-citation frequency were integrated into a single network, following [54]. We then ran the Clique Extracted Ontology (CliXO) algorithm [28], which identifies nested cliques at thresholds of gene-gene similarity that become progressively less stringent. The output of this algorithm was a directed acyclic graph of 4,766 cliques, with each clique representing a cellular subsystem. Code for constructing the ontotype matrix is available at https://github.com/michaelkyu/ontotype.

### Alignment of data-driven ontologies to GO

We next sought to assign labels to the terms in the data-derived ontology. We used the ontology alignment algorithm [27] present in the Data-Driven Ontology Toolkit (DDOT; https://github.com/michaelkyu/ddot) [55] with an FDR cutoff of 0.1 and 100 randomized iterations. For the 1,811 (38%) data-derived systems that contained a significant overlap with genes in a GO term (FDR < 0.1), these systems were labeled based on that matching GO term. The remaining novel 2,955 (62%) systems, cellular systems that did not align well to GO, were labeled with an alpha-numerical name: “CliXO” followed by a number.

### Identification of cellular subsystems using other systems-based approaches

GSA-SNP2 [23] and Gowinda [32] were used to identify statistically associated cellular subsystems (*P* < 0.05) using SNV *P* values from the original yeast GWAS [29]. The network of genes to GO terms is equivalent to the hierarchical structure and gene-to-term annotations used for ontotypes. For the GSA-SNP2 analysis, the input data consists of a tab-delimited two column text file in which one column is SNP IDs (rs numbers) and the other is *P* values from a GWAS. For the Gowinda analysis, the following command was used: java -Xmx4g -jar <path-to-gowinda>/Gowinda.jar—snp-file total_snps.txt—candidate-snp-file cand_snps.txt—gene-set-file goassociations_cg.txt—annotation-file annotation.gtf—simulations 100000—min-significance 1—gene-definition gene—threads 8—output-file results_gene_gene.txt—mode gene—min-genes 1.

### Machine learning models using random forests, mixed random forests and neural networks

Random forests from the R-source ranger package (version 0.11.2) [56] were used to regress phenotypic traits with 5-fold cross-validation. In each random forest, 1000 trees were used. Every tree was learned over a bootstrap sample of ontotypes. For predicting phenotypic outcomes using mixed random forests, ensembles of 1000 trees were used to build mixed random forest models [30] predicting each of the 36 yeast traits. The mixed random forest method is part of the LIMIX software package, which is available at https://github.com/PMBio/limix. For predictions using neural networks, sigmoid activation functions and 3 hidden layers were used. The number of nodes per hidden layer was as follows: 1000, 400 and 100. This method is part of the keras software package and is available at https://github.com/keras-team/keras. For 5-fold cross-validation, the original sample was randomly partitioned into 5 equally sized subsamples. Of the 5 subsamples, a single subsample was held out as the validation data for testing the regression model. The remaining 4 subsamples were used as training data. Then, the cross-validation process was repeated 5 times with each of the 5 subsamples used exactly once as the validation data. The R^2^ score for each model is the average R^2^ score across the 5 validation tests.

### Calculating feature importance, empirical *P* values and empirical FDRs

The importance of any input feature – ontotype, gene or SNV – in random forest-based models was generated using permutation tests as implemented in the ranger R package. The distribution of the importance under the null hypothesis of no association to the response is created by 100 replications of permuting the response, growing a random forest and computing the variable importance [57]. One is added to the numerator and denominator to avoid zero *P* values. Features with *P* < 0.01 across all 5 folds had a positive effect on predictive performance. The feature importance scores and *P* values for the well-predicted traits from both the random forest models and mixed random forest models are provided in S1 Table. In multiple figure panels, this analysis was the basis for the intensity of blue color for subsystems and red color for genes. To quantify the overlap of important features identified by the mixed random forest models compared to the random forest models, odds ratios were calculated using the 30 most important features. Fisher’s exact test was used to test for statistical significance (the significance of the deviation from a null hypothesis) of the resulting contingency tables. To generate empirical FDRs, a scrambled version of the ontotype matrix was used to build mixed random forest models as described above (i.e., 100 permutations and 5-fold cross-validation). From there, we determined how many features had a *P* value < 0.01 and used this value to calculate an empirical FDR.

### GO enrichment analysis on important gene or SNV features and mutants

GOstats (ver 2.48.0) and GSEABase (ver 1.44.0) were used to perform GO enrichment analysis on statistically important features (*P* < 0.01) from either gene-based or SNV-based models [58,59]. GO enrichment analysis was performed using all three branches of GO: MF, BP and CC.

### Validation of important systems related with growth phenotypes

To determine the impact of mutations in important systems, we compared the growth phenotype of strains with mutations in the systems of interest to strains with an equal number of mutations in randomly selected sets of genes outside said system. Random sets of genes were selected 1000 times. To test for statistically significant differences between the growth phenotypes, one-way ANOVA tests with Tukey’s correction for multiple comparisons were performed using the multcomp R package [60].

### Calculation of narrow-sense heritability

The proportion of phenotypic variance according to additive genetic variation, known as narrow-sense heritability (h^2^), can be estimated by calculating the proportion of the variance explained by SNVs: h^2^  = σ^2^_g_ / (σ^2^_g_+σ^2^_e_), where σ^2^_g_ and σ^2^_e_ are estimates of additive genetic and epistatic variances for the trait, respectively. These two values are estimated using the additive and epistatic relationship matrices calculated by the sommer R package [61]. These relationship matrices can be calculated using all SNVs or the set of SNVs that are used when making phenotypic predictions for the models (e.g., the 18,846 SNVs used for calculating the ontotype-based models).

### Quantification and statistical analysis

Statistical analyses were performed using R version 3.5.1. Detailed information regarding statistical tests (t-tests with Welch’s correction, one-way ANOVA test, *z*-score) used in this study have been provided in the figure legend or in the respective results or Materials and Methods section. Data are presented as points or boxplots. For all boxplots, the upper and lower hinges correspond to the first and third quartiles. The whiskers extend from the hinge to the largest and smallest values, no further than 1.5 x interquartile range from the hinge. Values beyond this range are shown as individual points. The center line indicates the median.

## Supporting information

S1 Fig Comparison of Random Forest, Mixed Random Forest and Neural Network Models Using Ontotypes as Features. (A) Scatter plot of R^2^ scores across 36 phenotypes in each model. RF, random forest; Mixed RF, mixed random forest. Five-fold cross- validation was examined using ontotypes as input features. (B) Odds ratios (B) and (C) statistical significance based on Fisher’s exact test comparing the overlay between the top 30 most important features from mixed random forest models and random forest models. (D) Scatter plot of R^2^ scores across 36 phenotypes in each model. NN, neural net; Mixed RF, Mixed random forest. Five-fold cross-validation was examined using ontotypes as input features.
(EPS)

S2 Fig Prediction Performance Using Mixed Random Forest Models for 36 Phenotypic Traits. Related to Figs 1 and 2. R^2^ values from five-fold cross-validation are averaged. Three types of input features are compared: ontotypes (A), genes (B) and SNVs (C).
(EPS)

S3 Fig Important Genes and Systems in Prediction of the Six Well-Predicted Traits. Related to Fig 2. The sets of genes identified by SNV- or gene-based models were translated to systems using GO enrichment analysis. The boxplots show the size distributions of these systems in comparison to systems identified directly by features in ontotype analysis.
(EPS)

S4 Fig Genetics and Mechanisms of Benomyl Sensitivity. Related to Fig 3. (A) Heatmap showing importance of the fifteen most important systems in predicting benomyl response. Similar to Fig 3A. (B) Subhierarchy of systems (circles) and genes (squares) related to benomyl response. Systems with red labels are among the fifteen most important features for prediction. Layout similar to Fig 3B but with feature importance for benomyl response on the left half and for normal YPD culture conditions on the right half. (C) Relationship between yeast growth and the number of mutated genes in the system “glucose transmembrane transporter activity.” Similar to Fig 3D.
(EPS)

S5 Fig Predictive Performance Characterization of Data-derived Systems. Related to Fig 5. (A) Phenotypic prediction performance using R^2^ score across 36 traits (y axis), shown for two alternative genetic analysis methods (x axis). R^2^ scores from five-fold validation were averaged. (B) Scatterplot showing R^2^ scores obtained for data-derived systems versus literature-derived systems in GO. Blue line is the regression line with 95% confidence intervals shown with the pale blue shadow. (C) R^2^ scores for individual phenotypic traits when using data-derived systems.
(EPS)

S6 Fig Genetics and Mechanisms of Copper Sensitivity using Data-Derived Systems. Related to Figs 3 and 5. (A) Hierarchy of data-derived systems and genes within the novel system CliXO10463. Layout similar to Fig 3B. (B) Relationship between yeast growth and number of mutated genes in CliXO8324. Similar to Fig 3D.
(EPS)

S7 Fig Genetics and Mechanisms of HU Sensitivity using Data-Derived Systems. Related to Figs 4 and 5. (A) Hierarchy of data-derived systems and genes within the “acid-thiol ligase” system (CliXO9394). Layout similar to Fig 4B. Systems labeled in blue are among the 30 most important for HU sensitivity. (B) Relationship between yeast growth and the number of mutated genes in CliXO9394. Similar to Fig 4C. (C) Relative change in sensitivity to HU in targeted single gene deletion mutants versus wild type. Plotted for each single gene deletion are the log2 ratios of growth under normal conditions relative to growth when treated with HU. Similar to Fig 4E.
(EPS)

S1 Table Feature importance scores and *P* values for the well-predicted traits from both the random forest models and mixed random forest models. (XLSX)

S2 Table Narrow-sense heritability scores for various models and phenotypes. (XLSX)

S3 Table List of genes most responsible for explaining heritability in the ontotype models and each gene’s ranked importance per phenotype. (XLSX)
